# Exploring the time-dependent regulatory potential of microRNAs in breast cancer cells treated with proteasome inhibitors

**DOI:** 10.1007/s12094-023-03349-5

**Published:** 2023-12-01

**Authors:** Katerina Katsaraki, Christos K. Kontos, Gerasimos Ardavanis-Loukeris, Alexandros A. Tzovaras, Diamantis C. Sideris, Andreas Scorilas

**Affiliations:** 1 Department of Biochemistry and Molecular Biology, Faculty of Biology, National and Kapodistrian University of Athens, Panepistimiopolis, 15701 Athens, Greece; 2 First Department of Medical Oncology, “Saint Savvas” General Anticancer Hospital of Athens, 11522 Athens, Greece

**Keywords:** Bortezomib, Carfilzomib, Small non-coding RNAs, Gene expression, Signaling pathways

## Abstract

**Purpose:**

Breast cancer (BrCa) is a predominant type of cancer with a disparate molecular nature. MicroRNAs (miRNAs) have emerged as promising key players in the regulation of pathological processes in BrCa. Proteasome inhibitors (PIs) emerged as promising anticancer agents for several human malignancies, including BrCa, inhibiting the function of the proteasome. Aiming to shed light on the miRNA regulatory effect in BrCa after treatment with PIs, we used two PIs, namely bortezomib and carfilzomib.

**Materials and methods:**

Four BrCa cell lines of distinct molecular subtypes were treated with these PIs. Cell viability and IC_50_ concentrations were determined. Total RNA was extracted, polyadenylated, and reversely transcribed. Next, the levels of specific miRNAs with a significant role in BrCa were determined using relative quantification, and their regulatory effect was assessed.

**Results:**

High heterogeneity was discovered in the levels of miRNAs in the four cell lines, after treatment. The miRNA levels fluctuate with distinct patterns, in 24, 48, or 72 hours. Interestingly, miR-1-3p, miR-421-3p, and miR-765-3p appear as key molecules, as they were found deregulated, in almost all combinations of cell lines and PIs. In the SK-BR-3 cell line, the majority of the miRNAs were significantly downregulated in treated compared to untreated cells, with miR-21-5p being the only one upregulated. Finally, various significant biological processes, molecular functions, and pathways were predicted to be affected.

**Conclusions:**

The diversity of pathways predicted to be affected by the diversity in miRNA expression after treatment with PIs paves the way for the recognition of new regulatory axes in BrCa.

**Supplementary Information:**

The online version contains supplementary material available at 10.1007/s12094-023-03349-5.

## Introduction

Breast cancer (BrCa) is the most frequent type of cancer in women, with an expected incidence of over 30% and 15% mortality for 2023 [1]. Novel treatment strategies have greatly improved the survival of patients as the therapeutic approach is personalized and decided based on the molecular characteristics of each patient. The hormone receptors (estrogen and progesterone) status, human epidermal growth factor receptor 2 (HER2) status, and the levels of the proliferation marker Ki-67 are the key components assessed for the stratification of patients into distinct molecular subtypes. Gene mutations in *BRCA1*, *BRCA2*, *PTEN*, *TP53*, and other genes as well as the expression of specific markers, such as PD-L1, contribute to an improved therapeutic strategy decision [2]. In recent years, numerous drugs have been approved for the treatment of BrCa patients, targeting unique molecules that contribute to the cancerous phenotype, such as Everolimus targeting mTOR and Abemaciclib targeting CDK4/6 [3].

Proteasome inhibitors (PIs) compose a significant class of drugs that regulate protein equilibrium, cell cycle progression, and other biological processes by inhibiting the function of the proteasome [4]. PIs bind to one of the catalytic subunits responsible for the proteasome chymotrypsin-like activity affecting tumor suppressors, cyclin-dependent kinases, metabolic pathways, and other key processes, and may eventually lead to apoptosis [5, 6]. The beneficial effect of PIs is under investigation for the last few years in a series of hematological malignancies and solid tumors revealing promising but, however, puzzling results, because of acquired resistance of cancer cells and side effects in patients. The role of PIs in BrCa has been investigated in numerous research studies and clinical trials focusing mainly on the triple-negative molecular subtype and metastatic BrCa, with a PI being given as a monotherapy or combinational therapy [6, 7]. Despite the fact that PIs are known to affect cell survival and other biological processes by affecting signaling pathways such as the JAKs/STATs and NF-κB pathways, little information exists about their role in BrCa.

MicroRNAs (miRNAs) belong to the class of small non-coding RNAs and have an average length of 22 nucleotides. They appear as fine regulators of gene expression, as well as molecules with a valuable biomarker utility and enormous potential in therapeutic interventions [8, 9]. miRNAs regulate numerous processes that take place in cells including survival, proliferation, differentiation, and metastasis, by sequence similarity with the mRNA target. Their fine tuning depends on their abundance in a specific topology and their involvement in both physiological and pathological states, such as cancer has been well documented [10]. miRNAs appear as key molecules in BrCa [11, 12]. Their role as biomarkers is commonly highlighted [13, 14], and their regulatory potential seems to affect cell viability and death, cell proliferation, tumor aggressiveness, and metastasis [15–17]. Their involvement in significant signaling pathways such as the MAPK/ERK [18], PI3K/AKT [19], NF-κB [20], and WNT/β-catenin [21] pathways has also been documented, and their interaction with long non-coding RNAs seems promising as well [22]. Lastly, they appear as molecules with great importance in BrCa therapy, as they have been proposed to contribute to BrCa cell sensitivity to treatment or chemoresistance [23, 24].

Given the significant role of miRNAs in the regulation of BrCa and the previously inadequately studied regulatory potential of PIs at a miRNA post-transcriptional level, we aimed at elucidating the levels of BrCa-significant miRNAs after treatment with PIs, as a first step to pave the way for the recognition and investigation of potential regulatory axes. In this study, specific deregulated miRNAs were identified, after the treatment of BrCa cell lines with PI, and the subsequent effect on biological processes, molecular functions, and pathways was explored. This was elucidated in four BrCa cell lines of distinct molecular subtypes after treatment with two PIs, bortezomib and carfilzomib. To the best of our knowledge, this is the first study that predicts the relative regulatory effect caused by the expression of numerous miRNAs in BrCa, after the treatment with PIs.

## Materials and methods

### Cell culture conditions

Four well characterized and of distinct molecular subtype BrCa cell lines were purchased from the American Type Culture Collection (ATCC^®^). The MCF-7 (Luminal A), BT-474 (Luminal B), SK-BR-3 (HER2-positive), and MDA-MB-468 (Triple Negative) cell lines were cultured according to the ATCC guidelines, in a humidified incubator at 37 °C and 5% CO_2_.

### Determination of the IC_***50***_ values and documentation of apoptosis after treatment with bortezomib and carfilzomib

Optimal seeding concentrations were determined for the four cell lines, in which the cells would proliferate at a consistent rate achieving a high number of cells in the final 72-hour time point. In the next step, two PIs, bortezomib (Velcade, PS-341) and carfilzomib (Kyprolis, PR-171) were used for the treatment of the four cell lines. Cell viability together with the inhibitor-induced cytotoxicity were assessed in 24, 48, and 72 hours by the Sulforhodamine B assay, using Sulforhodamine B (Invitrogen, Thermo Fisher Scientific), and the Trypan blue exclusion assay, using 0.4% (w/v) Trypan blue solution (Sigma-Aldrich, Merck). The IC_50_ value, which represents the half-maximal inhibitory concentration of each inhibitor, was determined for each combination of cell line and PI, after performing a wide-range screening of PIs concentrations between 1 and 100 nM. Apoptosis was assessed by the Caspase-3 activity colorimetric assay, following the manufacturer’s instructions (Elabscience, USA). Cell images were obtained with a Carl Zeiss^™^ Axio Vert.A1 Inverted Microscope (Carl Zeiss, Germany).

### Nucleic acid extraction, *RNA polyadenylation*, and reverse transcription of poly(A) RNA

Total RNA from treated and untreated cells in 24, 48, and 72 hours was extracted using the NucleoZOL (Macherey–Nagel, Germany) reagent and diluted in DEPC-treated H_2_O. The purity and concentration of total RNA were determined using a BioSpec-nano Micro-volume UV–Vis Spectrophotometer (Shimadju, Kyoto, Japan), and its integrity was assessed electrophoretically in an agarose gel.

Next, 200 ng of total RNA was in vitro polyadenylated using *E. coli* poly(A) polymerase (New England Biolabs, USA) and first-strand cDNA was synthesized, using an oligo-dT–adapter primer and the M-MLV reverse transcriptase (Invitrogen, Thermo Fisher Scientific). All reactions were performed according to the manufacturer’s instructions in a 96-well Veriti Thermal Cycler (Applied Biosystems, Thermo Fisher Scientific).

### Relative quantification of miRNA levels based on real-time qPCR

miRNAs with eminent roles in BrCa were explored in the bibliography and miRNA-specific primers were designed along with primers for the amplification of two reference genes, in order to assess their levels (Table S1). The small nucleolar RNAs *SNORD43* and *SNORD44* were used as reference genes for the relative quantification of miRNAs using the comparative C_T_ (2^−ΔΔCt^) method [25]. Real-time qPCR assays followed, using the forward-specific primers, a universal reverse primer, and KAPA^™^ SYBR^®^ FAST qPCR master mix (2X) (Kapa Biosystems Inc., Woburn, MA, USA), in a total reaction volume of 10 μL. Reactions were performed in a QuantStudio 5 Real-Time PCR System (Applied Biosystems, Thermo Fisher Scientific Inc.), according to the manufacturer’s protocol, and the prerequisites of the quantitative method were checked. The normalized levels of miRNAs in each sample were calculated using the geometric mean of *SNORD43* and *SNORD44* levels. The fold change of miRNA levels is presented as a log_2_ fold change, and a log_2_ fold change ≥ 1 and ≤ -1 was considered to be significant. Moreover, combinations of BrCa cell lines with PIs treatments, and miRNA expression were clustered using the Heatmapper tool to discover potential similarities between the expression of miRNAs in samples and the miRNA signature of samples [26]. The average linkage clustering method and the Euclidean distance measurement method were chosen for clustering.

### Prediction of miRNA targets and affected biological processes, molecular functions, and pathways

The experimentally validated and predicted targets of miRNAs, with significant differences in their expression levels after treatment, were explored using miRTarBase v.9.0, miRDB v.6.0, and TargetScanHuman v.8.0 databases [27–29]. Moreover, GO biological processes, GO molecular functions, as well as KEGG pathways in which the experimentally validated and predicted miRNA targets are involved, were identified by custom analysis with Metascape v.3.5 [30].

## Results

### Optimal seeding concentrations and IC_***50***_ values for the treatment of the BrCa cell lines

The optimal seeding concentration in which the cell cultures proliferated at a consistent rate, resulting in a high number of cells at the final 72-hour time point, was determined for each of the four cell lines. These concentrations were 6.00 × 10^4^ cells/mL for the MCF-7 cell line and 1.25 × 10^5^ cells/mL for the BT-474, SK-BR-3, and MDA-MB-468 cell lines.

**Fig. 1:**
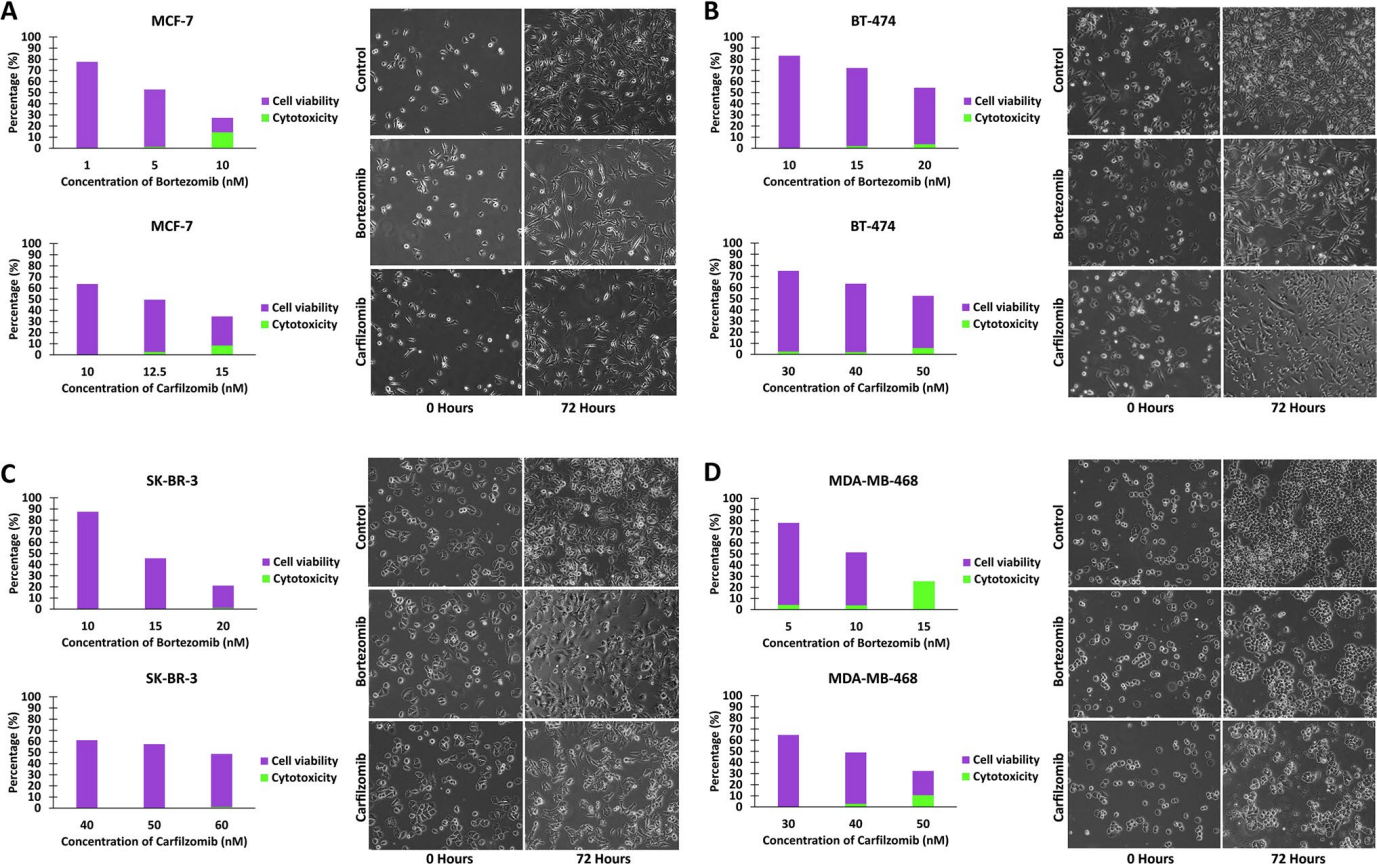
Cell viability and cytotoxicity after treatment with different concentrations of the two PIs, and cell culture images after treatment with the IC_50_ concentrations of the MCF-7 (**A**), BT-474 (**B**), SK-BR-3 (**C**), and MDA-MB-468 (**D**) cell lines

In the next step, a wide-range screening for the approximation of the IC_50_ values after the treatment of the four cell lines with both bortezomib and carfilzomib was performed (Fig. S1). This screening was performed in 96-well culture plates for 1, 10, and 100 nM of PI in all combinations of cell lines and PIs. Lastly, the IC_50_ values that indicate the amount of PI that is essential to inhibit the number of viable cells in half in 72 hours were determined, along with the percentage of drug-induced cytotoxicity (Fig. 1). The IC_50_ values for bortezomib and carfilzomib are 5 nM and 12.5 nM for the MCF-7 cell line, 20 nM and 50 nM for BT-474, 15 nM and 60 nM for SK-BR-3, 10 nM and 40 nM for MDA-MB-468, respectively. Moreover, approximately 50% of cell viability in treated cells can also be observed at 72 hours, in images from the respective cell cultures (Fig. 1). Finally, the presence of PI-induced apoptosis was documented (Fig. S2).

From these data, the MCF-7 cell line which belongs to the Luminal A molecular subtype appears to be more sensitive to the treatment with the two PIs in comparison to the other cell lines. Moreover, in the abovementioned results, treatments with bortezomib appear to be more dose-efficient in all treatment combinations in comparison to treatments with carfilzomib, as bortezomib appears effective in lower concentrations. Furthermore, the drug-induced cytotoxicity appears to be limited in the specified IC_50_ values.

### Expression patterns of miRNAs reflect the high heterogeneity of BrCa molecular subtypes

**Fig. 2:**
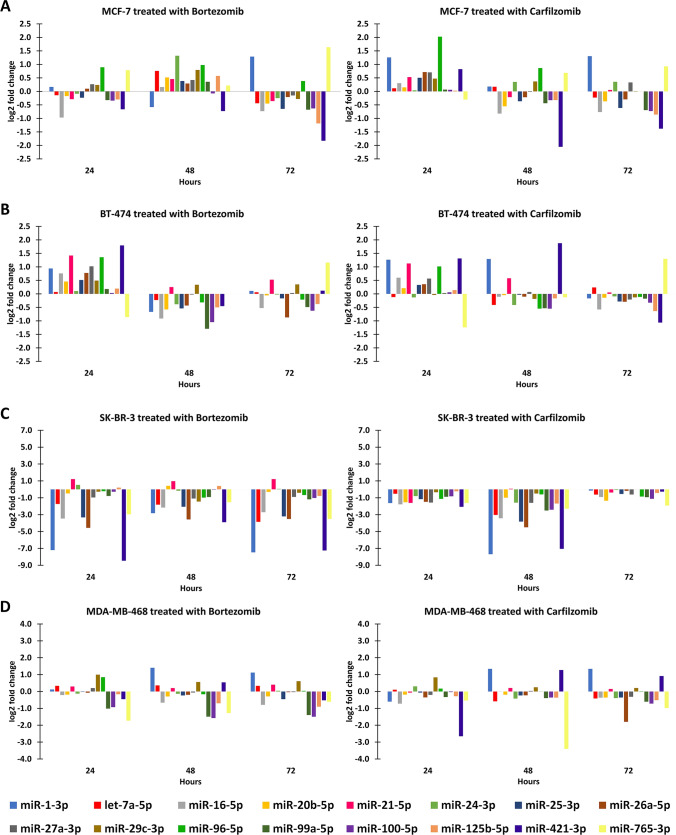
Expression levels of the studied miRNAs in 24, 48, and 72 hours after treatment with bortezomib or carfilzomib in the MCF-7 (**A**), BT-474 (**B**), SK-BR-3 (**C**), and MDA-MB-468 (**D**) cell lines

**Fig. 3:**
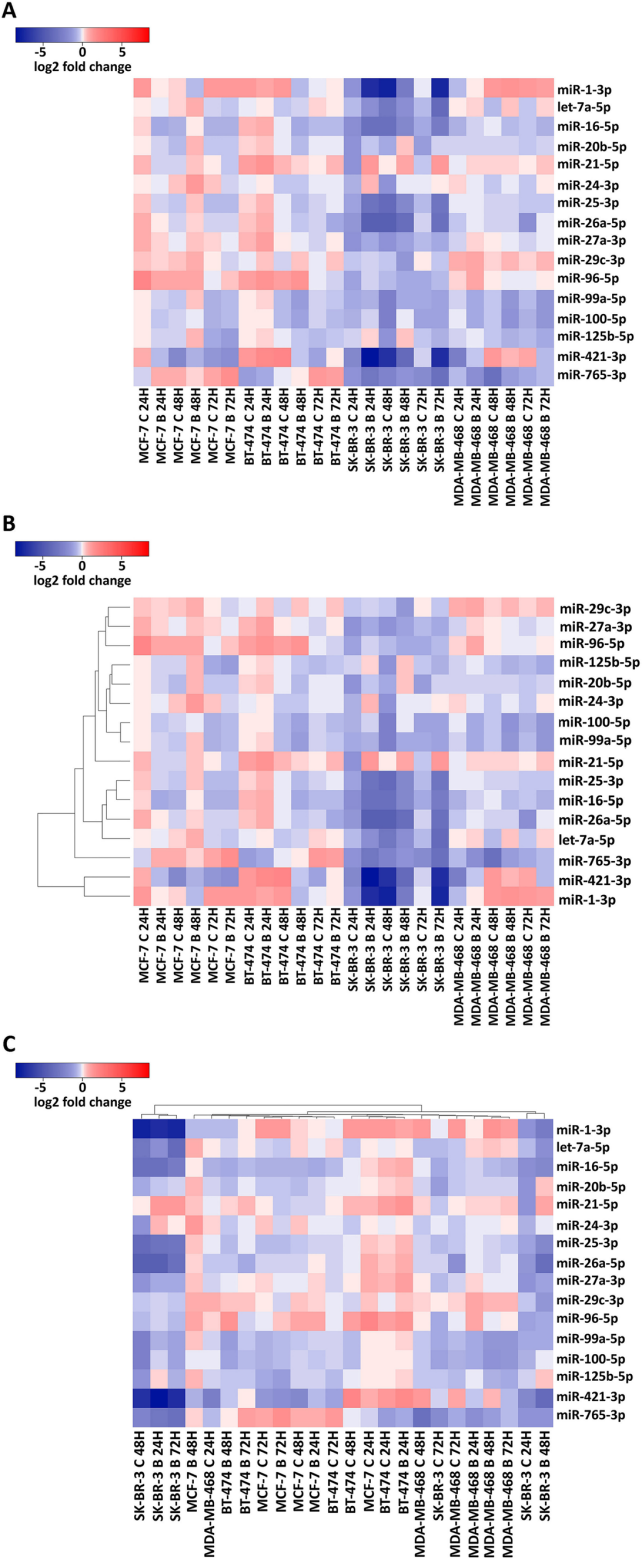
Expression levels of the studied miRNAs in all combinations of breast cancer cell lines and PIs without clustering (**A**), with clustering of the miRNA expression levels (**B**), and with clustering of the samples (**C**)

The relative levels of each miRNA were determined using the comparative C_T_ (2^−ΔΔCt^) method, with the geometric mean of *SNORD43* and *SNORD44* serving as a reference. Moreover, the specificity of each amplification product was checked with melt curve analysis. The normalized RQU or fold change values were expressed as log_2_ fold change for each combination of cell line and inhibitor (Fig. 2 and Fig. 3A).

The expression pattern of miRNAs seems to differ in each combination of cell line and PI which has been used for the relative treatment. Moreover, the expression of miRNAs appears highly heterogeneous over time with specific cases reaching peaks in 24, 48, or 72 hours, respectively, and a restoration of their levels being observed in prolonged time. For example, miR-96-5p levels present a peak in the 24-hour time point whereas miR-16-5p levels present a peak in the 48-hour time point in the BT-474 cells treated with bortezomib. Furthermore, a restoration of the levels of miR-96-5p can be observed in the 72-hour time point in the MCF-7 cells treated with carfilzomib. In many cases, the expression of each miRNA appears to be similar when comparing the two treatment strategies in a specific cell line. However, a limited time difference can be observed in the expression of most miRNAs between the treatments with the two PIs. In addition, the expression of numerous miRNAs, such as let-7a-5p and miR-21-5p in the MDA-MB-468 cell line, appears to not be affected by the treatments in the selected time points.

Interestingly, the majority of miRNAs seem to be considerably downregulated in the treated SK-BR-3 cells in comparison to the relative controls, for both inhibitors. The miR-21-5p appears as an exception in the SK-BR-3 cell line, as it is the only substantially upregulated miRNA after the treatment with bortezomib. Moreover, let-7a-5p, miR-16-5p, and miR-25-3p seem to be significantly downregulated only in the SK-BR-3 cell line. The substantially deregulated miRNAs in each combination of cell line and PI are summarized in Table 1. miR-1-3p, miR-421-3p, and miR-765-3p appear to be significantly deregulated in the majority of treatment combinations, a finding that potentially highlights a considerable role of these miRNAs in the mechanism of action of PIs. Besides these three cases, significantly deregulated miRNAs appear to be different in each combination of cell line and PI.

**Table 1:** Significantly deregulated miRNAs in each combination of breast cancer cell line and corresponding PI treatment

Cell line	Proteasome inhibitor	Significantly deregulated miRNAs
MCF-7	Bortezomib	miR-1-3p, miR-24-3p, miR-125b-5p, miR-421-3p, miR-765-3p
	Carfilzomib	miR-1-3p, miR-96-5p, miR-421-3p
BT-474	Bortezomib	miR-21-5p, miR-27a-3p, miR-96-5p, miR-99a-5p, miR-100-5p, miR-421-3p, miR-765-3p
	Carfilzomib	miR-1-3p, miR-21-5p, miR-96-5p, miR-421-3p, miR-765-3p
SK-BR-3	Bortezomib	miR-1-3p, let-7a-5p, miR-16-5p, miR-21-5p, miR-25-3p, miR-26a-5p, miR-27a-3p, miR-29c-3p, miR-99a-5p, miR-100-5p, miR-421-3p, miR-765-3p
	Carfilzomib	miR-1-3p, let-7a-5p, miR-16-5p, miR-20b-5p, miR-21-5p, miR-24-3p, miR-25-3p, miR-26a-5p, miR-27a-3p, miR-96-5p, miR-99a-5p, miR-100-5p, miR-125b-5p, miR-421-3p, miR-765-3p
MDA-MB-468	Bortezomib	miR-1-3p, miR-99a-5p, miR-100-5p, miR-765-3p
	Carfilzomib	miR-1-3p, miR-26a-5p, miR-421-3p, miR-765-3p

### miRNA expression clustering reveals substantial similarities and differences

In an effort to identify similarities in the expression of miRNAs, clustering was performed using the Heatmapper tool. Clustering of miRNA expression patterns uncovered the similarity in the expression of specific miRNAs in all samples (Fig. 3B). For example, miR-1-3p and miR-421-3p levels appear to derive in a similar time-dependent manner in each cell line studied. Moreover, miR-99a-5p and miR-100-5p, which are members of the same miRNA family and share a similar 5ʹ-seed region, appear to share similar expression patterns as well, among combinations of cell lines and the corresponding treatments.

Furthermore, the clustering of samples according to their miRNA expression patterns revealed the molecular similarity of specific combinations of cell line and PI used for the treatment (Fig. 3C). Interestingly, MCF-7 and BT-474 cell lines that correspond to Luminal A and Luminal B molecular subtypes are characterized by a higher similarity in the miRNA expression profiles. The SK-BR-3 and MDA-MB-468 cell lines that correspond to HER2-positive and triple-negative molecular subtypes appear considerably different regarding their molecular profile, as they are clustered in the two edges of the distribution.

### Predicted regulatory effect of miRNA targets resulting from the treatment of BrCa cell lines with bortezomib and carfilzomib

**Fig. 4:**
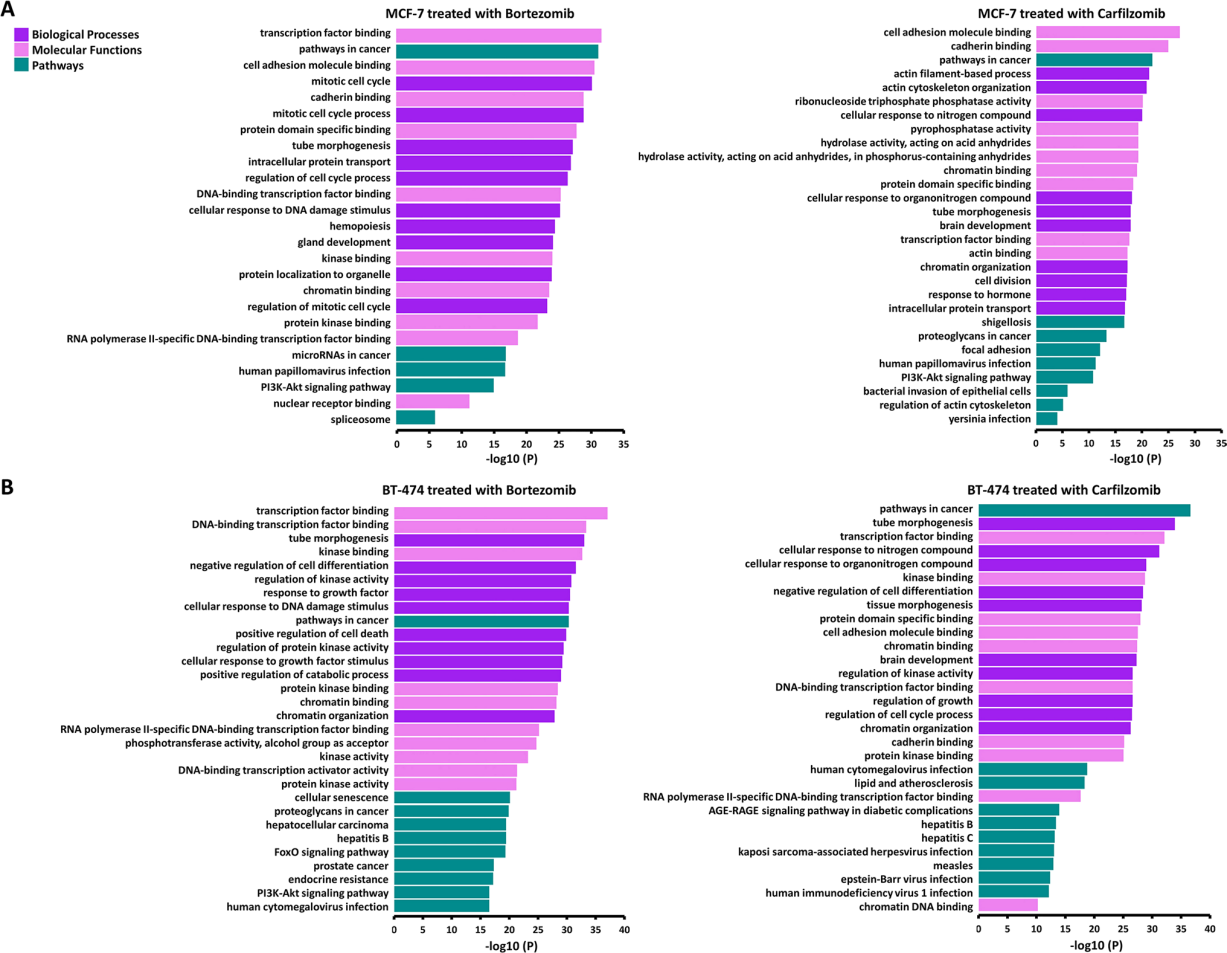

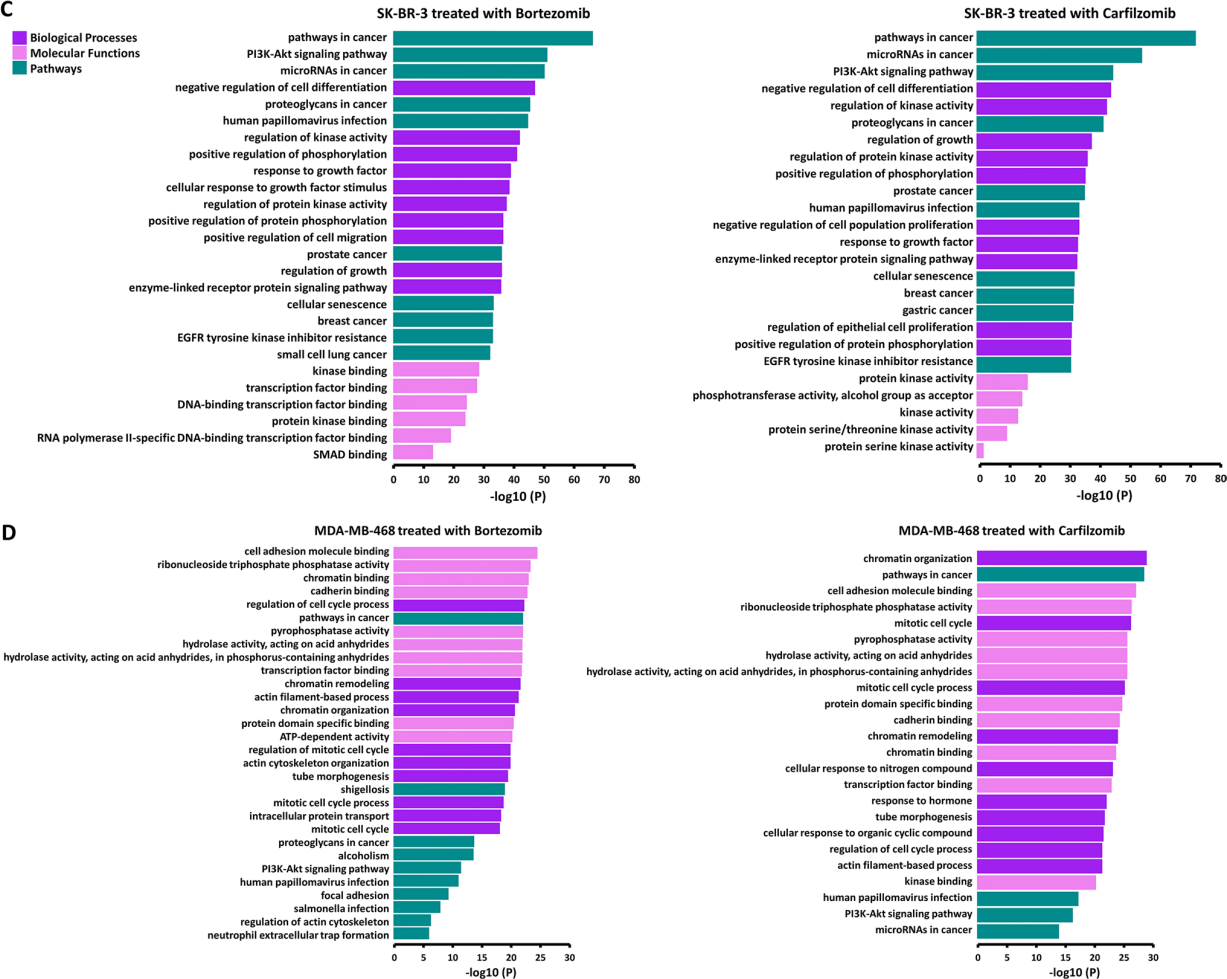
Τhe most enriched biological processes, molecular functions, and pathways in the MCF-7 (**A**), BT-474 (**B**), SK-BR-3 (**C**), and MDA-MB-468 (**D**) cell lines, after treatment with bortezomib and carfilzomib

The mRNA targets of the significantly deregulated miRNAs were explored (Table S2), in an effort to investigate the emerging regulatory effect in the BrCa cell lines, after the treatment with the PIs. The top 10 results from each category of affected biological processes, molecular functions, and pathways from the analysis of experimentally validated miRNAs targets, are illustrated for MCF-7 (Fig. 4A), BT-474 (Fig. 4B), SK-BR-3 (Fig. 4C), and MDA-MB-468 (Fig. 4D). Numerous functions, processes, and pathways seem to be altered such as the cell cycle process and cell differentiation which appear to be two of the most deregulated biological processes. Concerning molecular functions, treatment with the two PIs seems to mostly affect numerous molecular binding properties. Moreover, the pathways in cancer seem to be the most enriched pathway in every case and the PI3K/AKT pathway appears to be affected in almost all combinations studied. In addition, great similarities and discordances between cell lines, and the treatment with the two PIs in the same cell line, can be found in this level of analysis, as expected. Lastly, the respective analysis performed in predicted miRNA targets further revealed major discordances between the respective cell line and PI combinations.

## Discussion

The significant role of miRNAs in the regulation of BrCa and the remaining ambiguity of miRNA expression regulation by PIs urged us to explore the levels of miRNAs with considerable role in BrCa, after treatment with PIs, in order to recognize potential regulatory axes. Moreover, we chose to include in our study cell lines that belong to the four major molecular subtypes of BrCa in an effort to explore their heterogeneity. The chosen concentrations of the two PIs that were used in this study seem in accordance with previously published data from relevant experiments with a few BrCa cell lines [6, 31]. The MCF-7 cell line appeared to be the most sensitive of the four cell lines that were studied. Moreover, bortezomib appeared more dose-efficient in comparison to carfilzomib, and possessed, in most cases, a higher cytotoxic effect in higher concentrations.

The expression of the studied miRNAs is characterized by great heterogeneity. Moreover, as we study the expression of miRNAs in a broad time range, for transcriptomic purposes, limited or no change in the levels of specific miRNAs is observed. In addition, the restoration of their levels after the effect of the treatment does not allow us to clearly identify an upregulation or downregulation of miRNA levels in the course of time, in most cases. Therefore, a significant deregulation in the levels of each miRNA in treated cells in comparison to the untreated cells is mostly reported. This fact is not a limitation as our goal is to explore the potential regulatory effect after the respective treatments.

The similarity in the expression of specific miRNAs in treatments of a cell line with the two inhibitors may propose a similar mechanism of action, whereas discordances could indicate specific miRNAs as molecules with a controversial role. Interestingly, miR-1-3p, miR-421-3p, and miR-765-3p appear significantly deregulated, and with the same pattern according to clustering, in almost all combinations of treatments. This highlights their significant regulatory impact as they have been found to affect key signaling pathways such as the MAPK, and the NF-κB, and regulate BrCa cell proliferation, tumor growth, metastasis, and chemosensitivity by multiple mechanisms of action [20, 32, 33]. Furthermore, miR-421-3p appears to promote BrCa progression as well as to inhibit BrCa metastasis in another context [17, 34]. Therefore, the study of the regulatory potential of these three miRNAs could unravel novel insight for the mechanism of action of PIs. Three other miRNAs, let-7a-5p, miR-16-5p, and miR-25-3p, appear to be significantly downregulated, and with the same pattern according to clustering, only in the SK-BR-3 cell line. This observation may indicate a fine regulation by these miRNAs in the HER2-positive molecular subtype. However, even though these miRNAs appear as important biomarkers for BrCa [13], and molecules with great regulatory potential, relative scientific literature about their role in HER2-positive BrCa is extremely limited [35]. The other significantly deregulated miRNAs appear to possess a more molecular subtype-specific biomarker utility and regulatory potential, as their levels are considerably deregulated in more specific combinations of BrCa cell line and PI [16, 19, 22, 36–38].

Interestingly, the majority of miRNAs seem to be downregulated in the treated SK-BR-3 cells in comparison to the relative controls after treatment with both inhibitors. This appears as an interesting topic requiring further research, as it may reveal a specific mechanism of action of PIs in the HER2-positive molecular subtype. In addition, miR-21-5p appears to be an exception, as it is the only significantly and consistently upregulated miRNA in the SK-BR-3 cell line after treatment with bortezomib. The high levels of this miRNA have been documented to regulate malignant BrCa phenotypes and drug resistance [39, 40]. Therefore, in the context studied, the upregulated miR-21-5p expression after treatment with bortezomib may signify the activation of a bortezomib resistance pathway.

The clustering of samples in relation to the miRNA expression uncovered the molecular similarity of Luminal BrCa molecular subtypes and a high miRNA molecular heterogeneity of the HER2-positive and triple-negative subtypes. This observation validates the importance of miRNAs as biomarkers and molecules with significant regulatory roles with an impact on the molecular heterogeneity of this type of cancer. In addition, according to clustering, miR-99a-5p and miR-100-5p appear to possess similar expression patterns. This observation is important for the significance of the aforementioned results as these two miRNAs belong to the same miRNA family, and therefore, share the same 5ʹ-seed sequence. Therefore, a similar expression in the samples of the study indicates a similar regulatory effect in the studied combinations of BrCa molecular subtypes and treatments.

The regulatory potential of significantly deregulated miRNAs after treatment of BrCa cell lines with PIs uncovered the importance of this type of treatment and its promising therapeutic potential. As revealed from the corresponding analysis, numerous significant pathways, processes, and functions seem to be altered, with the pathways in cancer being the most enriched pathway in every case. Furthermore, the effect of treatments with PIs seems to affect molecular binding properties and to majorly regulate the cell cycle process and cell differentiation. The aforementioned data highlight the significance of treatment with PIs at the miRNA level and subsequently the importance of miRNA regulatory potential in cells.

The specific transcriptional effect of PIs remains largely undiscovered. Most studies focus on multiple myeloma treatment and refer to specific cell properties being affected. Prompted by these properties, specific miRNAs have emerged as regulatory players. For example, miR-145-3p appears to target *HDAC4*, promoting autophagy and enhancing bortezomib sensitivity in multiple myeloma [41]. Another study indicates miR-497 as a regulator of multiple myeloma cell growth and sensitivity to bortezomib, by the regulation of *BCL2* [42]. The effect of PIs on the regulatory potential of miRNAs has not been studied in the context of BrCa, with the only exception being a study that highlights let-7a-5p as an indicator of bortezomib sensitivity [43]. Current bibliography elucidates mainly cell properties and pathways affected after the treatment of BrCa cells, with the PI being provided as a monotherapy or part of combinational therapy, with no reference to miRNAs. Therefore, the innovation of our study is the investigation of the effect of PIs on miRNA expression in BrCa, and the predicted regulatory effect. The investigation of the regulatory effect of miRNAs in different BrCa molecular subtypes is another dynamic innovation with potential applications in the clinic. In addition, relative research for histological types of BrCa could unravel promising results [44]. Furthermore, we chose to investigate the miRNAs levels at numerous time points, in support of the previous bibliography [45], and uncovered the kinetics of the potential regulatory effect of miRNAs. In addition, the heterogeneity in the miRNA expression patterns between the different cell lines that belong to distinct molecular subtypes of BrCa highlights the importance of incorporating the parameter of molecular subtype in relative studies.

The investigation of the regulatory potential of miRNAs with universally deregulated levels in the studied combinations, such as miR-1-3p, as well as of miRNAs with molecular subtype-specific deregulated levels, such as let-7a-5p, could uncover novel mechanisms of action both for miRNAs and PIs in BrCa. Furthermore, the phenomenon of time-dependent differential expression of miRNAs in contrast to constantly downregulated or upregulated levels of miRNAs, such as miR-21-5p after treatment of the SK-BR-3 cell line with bortezomib, appears extremely intriguing. This observation may lead to significant results as a constant deregulation of miRNA levels, up to 72 hours post-treatment, may indicate a molecule as a key player in significant cellular processes and properties, such as the cell cycle and cell sensitivity. Moreover, such a constant expression pattern after treatment indicates specific miRNAs as candidate biomarkers.

In light of the above, and along with the importance of the post-transcriptional miRNA regulation, the validation of regulatory mRNA–miRNA axes after the treatment with PIs is of major importance, to propose interactions with great significance for this type of cancer, and to investigate in depth the therapeutic utility of PIs in BrCa.

### Supplementary Information

Below is the link to the electronic supplementary material.

Supplementary file1 (TIF 1683 KB)

Supplementary file2 (TIF 220 KB)

Supplementary file3 (DOCX 16 KB)

Supplementary file4 (DOCX 41 KB)

Supplementary file5 (XLSX 1829 KB)

## Data Availability

All data produced in the current study are available from the corresponding author upon reasonable request.

## Code availability

The R codes used to visualize the results of functional enrichment analysis are available from the corresponding author upon reasonable request.
