# Supplementary material for: Exploring the time-dependent regulatory potential of microRNAs in breast cancer cells treated with proteasome inhibitors
Source: Clin Transl Oncol. 2023 Dec 1;26(5):1256–67. doi: 10.1007/s12094-023-03349-5 (PMC11026233; doi:10.1007/s12094-023-03349-5)
Supplement: Supplementary file 3 — Supplementary file3 (DOCX 16 KB) [file 12094_2023_3349_MOESM3_ESM.docx]

**Supplementary Figure Legends**

**Fig. S1.** Cell viability of MCF-7 **(A)**, BT-474 **(B)**, SK-BR-3 **(C)**, and MDA-MB-468 **(D)** cell lines in 24, 48, and 72 hours after treatment with 1, 10, and 100 nM of bortezomib or carfilzomib.

**Fig. S2.** Caspase-3 activity in the studied cell lines, after treatment with bortezomib or carfilzomib.
