# Supplementary material for: Exploring the time-dependent regulatory potential of microRNAs in breast cancer cells treated with proteasome inhibitors
Source: Clin Transl Oncol. 2023 Dec 1;26(5):1256–67. doi: 10.1007/s12094-023-03349-5 (PMC11026233; doi:10.1007/s12094-023-03349-5)
Supplement: Supplementary file 4 — Supplementary file4 (DOCX 41 KB) [file 12094_2023_3349_MOESM4_ESM.docx]

**Suppl. Table 1**. Features of the primers used for the amplification of reference small nucleolar RNAs and miRNAs.

| **Target** | **Primer sequence (5′ 🡪 3′)** | **Length (nt** ^a^**)** |
| --- | --- | --- |
| *SNORD43* | ACTTATTGACGGGCGGACA | 19 |
| *SNORD44* | AGCAAATGCTGACTGAACATGA | 22 |
| miR-1-3p | TGGAATGTAAAGAAGTATGTATAAAAAA | 28 |
| let-7a-5p | TGGGATGAGGTAGTAGGTTGTATAG | 25 |
| miR-16-5p | TAGCAGCACGTAAATATTGGCG | 22 |
| miR-20b-5p | CAAAGTGCTCATAGTGCAGGTAGAA | 25 |
| miR-21-5p | GTAGCTTATCAGACTGATGTTGAAA | 25 |
| miR-24-3p | GCTCAGTTCAGCAGGAACAGAA | 22 |
| miR-25-3p | ATTGCACTTGTCTCGGTCTGA | 21 |
| miR-26a-5p | GGTTCAAGTAATCCAGGATAGGCTA | 25 |
| miR-27a-3p | CACAGTGGCTAAGTTCCGCA | 20 |
| miR-29c-3p | GCTAGCACCATTTGAAATCGGTTA | 24 |
| miR-96-5p | TGGCACTAGCACATTTTTGCTAAA | 24 |
| miR-99a-5p | AACCCGTAGATCCGATCTTGTGA | 23 |
| miR-100-5p | ACCCGTAGATCCGAACTTGTGA | 22 |
| miR-125b-5p | TCCCTGAGACCCTAACTTGTGAA | 23 |
| miR-421-3p | AACAGACATTAATTGGGCGCAA | 22 |
| miR-765-3p | GGAGGAGAAGGAAGGTGATG | 20 |
| Universal reverse primer | GCGAGCACAGAATTAATACGAC | 22 |

^a^ nucleotides.
